# Association Between Phase Angle and Sarcopenia in Patients Undergoing Peritoneal Dialysis

**DOI:** 10.3389/fnut.2021.742081

**Published:** 2021-09-24

**Authors:** Jun Young Do, A. Young Kim, Seok Hui Kang

**Affiliations:** Division of Nephrology, Department of Internal Medicine, College of Medicine, Yeungnam University, Daegu, South Korea

**Keywords:** peritoneal dialysis, sarcopenia, muscle mass, handgrip strength, bioimpedance

## Abstract

**Introduction:** There is limited data on the association between phase angle (PhA) and sarcopenia using both muscle strength and muscle mass in patients undergoing peritoneal dialysis (PD). We aimed to evaluate the association between PhA and sarcopenia in patients undergoing PD.

**Methods:** We enrolled prevalent patients undergoing PD (*n* = 200). The patients were divided into tertiles based on their PhA level: low (*n* = 66; 1.9–4°), middle (*n* = 68; 4.1–4.9°), and high tertiles (*n* = 66; 5–8°). PhA was measured by a bioimpedance analysis. Handgrip strength (HGS) was measured in all the patients. Body compositions were measured by dual energy x-ray absorptiometry (DXA).

**Results:** Handgrip strength (HGS) and/or lean mass indices showed poorer trends in the low tertile than in the other tertiles. PhA was positively associated with HGS and/or muscle mass index. Multivariate analyses showed that the patients in the low tertile had an odds ratio of 9.8 (*p* = 0.001) and 52.79 (*p* < 0.001) for developing sarcopenia compared with those in the middle and high tertiles, respectively. Subgroup analyses using these variables yielded results similar to those from the total cohort.

**Conclusion:** This study demonstrated that PhA is independently associated with muscle mass, strength, and sarcopenia in patients undergoing PD. This result suggests that PhA can be used as a valuable and simple predictor for identifying patients undergoing PD who are at risk of sarcopenia.

## Introduction

Sarcopenia is one of the most important complications in patients undergoing peritoneal dialysis (PD), with a prevalence of 8–15.5% (1–3). Sarcopenia is a progressive and generalized muscle disorder associated with increased falls, fractures, physical disability, and mortality (4), and it is classically defined as a combination of low muscle mass and low strength (4–6). Muscle strength is not affected by muscle mass alone, and the association between muscle mass and strength is not linear (6). This leads to the application of two indicators to diagnose sarcopenia in clinical practice. Sarcopenia was originally considered as results of the aging process.

However, recent studies have shown that inflammatory processes, such as malignancy and organ failure, can give rise to sarcopenia (4). Sarcopenia in patients undergoing PD is caused by various factors such as anorexia, uremic toxin, volume overload, or peritoneal dialysate (7), and it is strongly associated with the risk of falls, fractures, disability, and decreased cognition (8, 9). Two previous studies on patients undergoing dialysis have reported a hazard ratio of 1.93 and 2.92, respectively, for mortality in patients with sarcopenia than those without sarcopenia (10, 11). Pereira et al. included 287 patients with non-dialysis chronic kidney disease, among whom 17 had sarcopenia, and reported a hazard ratio of 3.02 in patients with sarcopenia compared with those without sarcopenia (12). Interventions, such as nutritional supplementation or exercise, can be applied to patients who are at high risk of sarcopenia. Some studies have shown favorable effects on protein homeostasis, exercise tolerance, quality of life, dialysis adequacy, and physical performance through nutritional support or exercise (13, 14). These findings reveal that the early diagnosis of sarcopenia can be useful in improving the prognosis of patients undergoing dialysis who have a high risk of sarcopenia. However, diagnostic methods may be difficult to apply to all patients. Recent guidelines have shown the usefulness of questionnaires, such as SARC-F, as a screening method. Still, the method has a very low positive predictive value in patients undergoing PD (4, 15). This limitation may require additional indicators to predict sarcopenia, especially the direct or indirect evaluation of muscle mass.

The bioimpedance analysis (BIA) is a popular body composition analysis technique and is well-validated for use in the general population. It is a non-invasive method for assessing the resistance and reactance in various regions and frequencies (generally between 1 and 1,000 kHz). The impedance measured using this device shows the bioelectrical characteristics of patients. Bioimpedance at currents of various frequencies is used to calculate body composition, estimated from regression equations derived from a healthy population (16). Recent guidelines recommend estimating muscle mass using a bioimpedance analysis (BIA) to diagnose sarcopenia (4). However, the body composition calculated from a BIA might not be accurate in populations with various diseases, especially volume-dependent patients such as those undergoing dialysis. A previous study showed a significant bias between muscle mass measurements using a BIA and the standard method in volume-dependent patients (17). The National Kidney Foundation-Kidney Disease Outcomes Quality Initiative (NKF-DOQI) guidelines reported a lack of evidence to recommend measuring body composition using a BIA. The guidelines also stated that body composition measurement data obtained using a BIA should be interpreted cautiously in a patient undergoing PD (18). Therefore, phase angle (PhA) as raw data with no regression equation can be an alternative method to predict various conditions. The PhA is regarded as a biological marker of cellular health, and the association between PhA and malnutrition or mortality in non-dialysis patients is well-known. Some studies showed the association between PhA and mortality or malnutrition in patients undergoing PD. These studies demonstrated a positive association between PhA and nutritional indices, such as subjective global assessment measurement, serum albumin, total protein, creatinine, blood urea nitrogen, prealbumin, and a Geriatric Nutritional Risk Index (GNRI) in patients undergoing PD (19–22). Furthermore, the associations with residual renal function, lean mass index, or mortality were obtained from previous studies (22, 23). However, most malnutrition indices in these studies were defined by body composition measurements from a BIA or an incomplete definition for sarcopenia (19–23). There were few data on the association between PhA and sarcopenia using both muscle strength and muscle mass. Although volume overloading can lead to the overestimation of fat-free mass by dual energy x-ray absorptiometry (DXA), the NKF-DOQI guidelines recommend that body composition measurement by DXA is reasonable in patients undergoing PD as it remains the gold standard for measuring body composition (18, 24). In addition, a BIA can be considered an alternative method to predict muscle mass in the guidelines for diagnosing sarcopenia. Although both DXA and BIA can be influenced by volume status, DXA has been validated and performed for direct measurements compared with a BIA. The European Working Group on Sarcopenia in Older People guidelines recommends DXA as a preferred method to diagnose sarcopenia (6). Muscle mass measurements using a more accurate method such as DXA, rather than BIA and muscle strength would be essential to evaluate the association between PhA and sarcopenia in patients undergoing PD. Thus, we aimed to evaluate the association between PhA and sarcopenia in patients undergoing PD.

## Methods

### Study Population

This study was retrospective and cross-sectional, and it used data from a tertiary medical center. In our center, handgrip strength (HGS), body composition measurement using DXA, and BIA measurements for volume status were routinely evaluated for all PD patients, followed by the outpatient department from September 2017. All the patients undergoing PD were informed of the necessity of evaluating sarcopenia, and the evaluation was performed if a patient agreed to the measurement. Three measurements were simultaneously performed on the same day of the peritoneal equilibration test at 6–12 month intervals. A trained nurse performed all the measurements during the study period. We evaluated these indicators, which have been used for patient management and advice. We planned analyses using data from the patients undergoing PD who also underwent all HGS, BIA, and DXA measurements on the same day from September 2017 to November 2020. Therefore, the size of the sample was not determined. There were 214 prevalent patients undergoing PD between September 2017 and November 2020, among whom 14 were excluded because of missing data (*n* = 8), the inability to ambulate, or having an amputated limb (*n* = 6). Therefore, 200 patients undergoing PD were included in this study. We used the most recent data from the three measurements on the same day if a patient had multiple measurements at two or more different time points. Finally, the patients were divided into tertiles based on the level of PhA as follows: low, middle, and high tertiles. The study was approved by the Institutional Review Board of Yeungnam University Medical Center (approval no: 2020-06-002). The board waived the need to obtain informed consent because the records and information of the participants were anonymized and de-identified prior to the analysis. The study was conducted ethically in accordance with the Declaration of Helsinki of the World Medical Association.

### Baseline Variables

Baseline data on age, sex, presence of diabetes mellitus (DM), dialysis modality, dialysis vintage (months), body mass index (kg/m^2^), weekly Kt/V_urea_, C-reactive protein (mg/dl), 4-h dialysate-to-plasma creatinine concentration ratio, urine volume (ml/day), edema index, serum calcium (mg/dl), phosphorus (mg/dl), sodium (mEq/L), potassium (mEq/L), and albumin (g/dl) levels were collected. DM was defined as a patient-reported history and a medical record of a DM diagnosis or medication. Weekly Kt/V_urea_ was calculated using 24-h urine and dialysate as previously published (25). Four-hour dialysate-to-plasma creatinine concentration ratio (DP4Cr) was evaluated using a modified 4.25% peritoneal equilibration test, and the level was calculated using the creatinine level of the drained dialysate 4 h after injection per the blood creatinine level. The edema index was defined as extracellular water/total body water from BIA measurements.

### Assessment of Nutritional Markers

PhA was measured using a multi-frequency BIA (InBody 770; InBody, Seoul, Korea). The value was calculated using an angle value of the time delay between the voltage waveform at 50 kHz and the current waveform. Briefly, the peritoneal dialysate was drained from the abdomen prior to measurement. Each subject was clothed with a light gown, and the bladder was emptied. Measurements were performed after rest for 5 min in the erect position. Eight electrodes were placed, two for each foot and two for each hand, with the patient in the erect position. Using reactance (Xc) and resistance (R) obtained from the BIA at 50 kHz, PhA was estimated by the following formula: PhA (°) = arctangent (Xc/R) × (180/p).

HGS was measured in all the patients. Each patient performed three trials with the dominant hand using a digital dynamometer (Takei 5401; Takei Scientific Instruments Co., Ltd., Niigata, Japan). The maximum strength measured over the three trials was recorded. Body compositions were measured using a DXA system (Hologic, Madison, WI, United States). The total lean mass (LM), appendicular lean mass (ALM), and total fat mass (FM) were estimated using this DXA system. The ALM was calculated using the sum of the lean masses of both extremities. The index values were defined as the value per height square. The visceral fat area (VFA, cm^2^) was measured from the BIA, and a previous study showed a strong correlation between measurements from the BIA and those from standard methods (26). The normalized protein nitrogen appearance (nPNA, g/kg/day) and the Geriatric Nutritional Risk Index (GNRI) were calculated from previously described equations (27, 28).

Sarcopenia was defined using cut-off values from the Asian Working Group for Sarcopenia (5). Patients with low muscle mass (ALM index <7 kg/m^2^ for men and <5.4 kg/m^2^ for women by DXA) and low HGS (<26 kg for men and <18 kg for women) were classified as having sarcopenia.

### Statistical Analysis

The data were analyzed using the statistical software IBM SPSS Statistics version 25 (SPSS Inc., Chicago, IL, United States). Categorical variables were expressed as counts (percentages). Continuous variables were expressed as mean ± SD or SE. For continuous variables, means were compared by one-way ANOVA, followed by a Bonferroni *post-hoc* comparison and an analysis of covariance for multivariate analyses. The correlation between two continuous variables was assessed by Pearson's or partial correlation analysis. Linear or logistic regression analyses were performed to assess the independent predictors of nutritional indices or sarcopenia. A multivariate analysis was adjusted for age, sex, the presence of DM, BMI, urine volume, and edema index. The area under the receiver operating characteristic curve (AUROC) was used to calculate the probability to predict sarcopenia, cutoff values, sensitivity, and specificity. The best cutoff value was calculated using the Youden index in the AUROC. The MedCalc version 11.6.1.0 software (MedCalc, Mariakerke, Belgium) was used for the AUROC. The level of statistical significance was set at *p* < 0.05.

## Results

### Clinical Characteristics of the Participants

The PhA intervals in the low, middle, and high tertiles were 1.9–4, 4.1–4.9, and 5–8°, respectively. The patients in the high tertile were younger than those in the other tertiles, and the proportion of female sex or DM was lowest in the high tertile. BMI, urine volume, and serum albumin levels were higher in the high tertile than in the other tertiles (Table 1). Among the patients, those in the high tertile had the lowest edema index. There were no significant differences in the use of automated PD, dialysis vintage, and weekly Kt/V_urea_ and the levels of C-reactive protein, DP4Cr, calcium, phosphorus, sodium, and potassium among the three tertiles.

**Table 1:** Clinical characteristics of the participants.

	**Total**	**Low tertile**	**Middle tertile**	**High tertile**	***p***
	**(*n* = 200)**	**(*n* = 66)**	**(*n* = 68)**	**(*n* = 66)**	
Age (years)	55.5 ± 12.2	59.4 ± 11.7	57.3 ± 11.2	49.7 ± 11.9^*^^#^	<0.001
Sex (men)	114 (57.0%)	30 (45.5%)	35 (51.5%)	49 (74.2%)	0.002
Diabetes mellitus (%)	99 (49.5%)	41 (62.1%)	37 (54.4%)	21 (31.8%)	0.001
Automated peritoneal dialysis	58 (29.0%)	14 (21.2%)	21 (30.9%)	23 (34.8%)	0.206
Dialysis vintage (months)	57.8 ± 53.2	63.1 ± 52.3	55.4 ± 52.2	55.1 ± 55.3	0.624
Body mass index (kg/m^2^)	24.7 ± 3.8	24.1 ± 3.1	24.1 ± 3.8	25.8 ± 4.1^*^^#^	0.011
Weekly Kt/Vurea	1.92 ± 0.46	1.93 ± 0.43	1.86 ± 0.43	1.98 ± 0.52	0.382
C-reactive protein (mg/dL)	0.57 ± 1.26	0.62 ± 1.42	0.66 ± 1.47	0.42 ± 0.75	0.491
DP4Cr	0.66 ± 0.13	0.69 ± 0.16	0.64 ± 0.11	0.65 ± 0.12	0.090
Urine volume (ml/day)	430 ± 603	361 ± 574	236 ± 385	697 ± 717^*^^#^	<0.001
Edema index	0.400 ± 0.013	0.412 ± 0.009	0.400 ± 0.007^*^	0.387 ± 0.009^*^^#^	<0.001
Serum calcium (mg/dL)	8.3 ± 0.9	8.3 ± 0.9	8.3 ± 1.0	8.3 ± 1.0	0.980
Serum phosphorus (mg/dL)	4.9 ± 1.4	4.7 ± 1.4	5.0 ± 1.3	5.0 ± 1.4	0.599
Serum sodium (mEq/L)	136 ± 4	136 ± 4	136 ± 4	137 ± 3	0.342
Serum potassium (mEq/L)	4.5 ± 0.7	4.5 ± 0.8	4.6 ± 0.6	4.6 ± 0.6	0.428
Serum albumin (g/dL)	3.6 ± 0.5	3.3 ± 0.5	3.6 ± 0.4^*^	3.8 ± 0.4^*^^#^	<0.001

*Data are expressed as mean ± SD for continuous variables and as numbers (percentages) for categorical variables. P-values were tested using a one-way ANOVA, followed by Bonferroni's post-hoc comparison for continuous variables and Pearson's χ^2^ or Fisher's exact tests for categorical variables*.

*DP4Cr, 4-h dialysate-to-plasma creatinine concentration ratio*.

* *p < 0.05, compared with low tertile and*

# *p < 0.05, compared with middle tertile*.

### Comparison of Nutritional Indices According to PhAs

**Figure 1:**
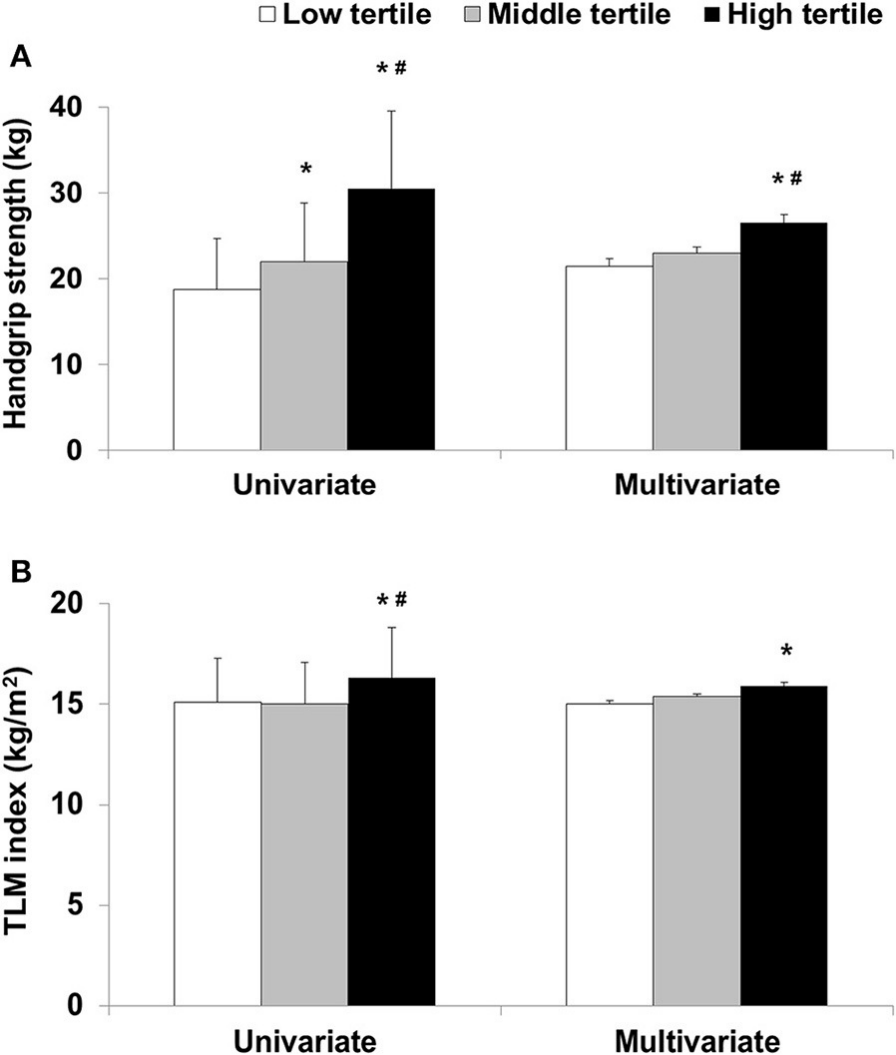
**(A)** Handgrip strength or **(B)** total lean mass (TLM) index according to phase angle tertile. ^*^p < 0.05 compared with low tertile and ^#^*p* < 0.05 compared with middle tertile.

In the univariate analyses, the values of HGS in the low, middle, and high tertiles were found to be 18.7 ± 6, 22 ± 6.8, and 30.5 ± 9 kg, respectively (*p* < 0.001). In the multivariate analyses, HGS values in the low, middle, and high tertiles were found to be 21.4 ± 0.9, 23 ± 0.7, and 26.5 ± 1 kg, respectively (*p* = 0.006, Figure 1). In the multivariate analyses, total LM index, and VFA were 15 ± 0.2 kg/m^2^ and 92.8 ± 3.4 cm^2^ in the low tertile, 15.4 ± 0.1 kg/m^2^ and 82.9 ± 2.6 cm^2^ in the middle tertile, and 15.9 ± 0.2 kg/m^2^ and 57.2 ± 3.7 cm^2^ in the high tertile, respectively (Table 2) (*p* =0.027 for total LM index and *p* < 0.001 for VFA). HGS was greatest in the patients in the high tertile. The total LM index was lower in the patients in the low tertile than those in the high tertile. VFA was lowest in the patients in the high tertile. Among the patients, those in the low tertile had the lowest nPNA. The ALM index and GNRI were lowest in the patients in the low tertile, but the difference was not statistically significant. PhA, as a continuous variable, was positively associated with HGS, total LM index, ALM index, total FM index, nPNA, and GNRI (Supplementary Table 1). The partial correlation adjusted for covariates showed positive associations with HGS, total LM index, ALM index, and nPNA, and showed inverse associations with total FM index and VFA. Linear regression analyses also showed positive associations between PhAs and both HGS and the ALM index as two indicators of sarcopenia in univariate and multivariate analyses (Supplementary Table 2).

**Table 2:** Comparison of muscle mass indices and nutritional and physical activity markers according to the tertiles of phase angle.

	**Univariate**				**Multivariate**			
	**Low tertile**	**Middle tertile**	**High tertile**	***p***	**Low tertile**	**Middle tertile**	**High tertile**	***p***
ALM index (kg/m^2^)	5.9 ± 1.1	6.0 ± 1.1	7.0 ± 2.9^*^^#^	<0.001	6.0 ± 0.3	6.2 ± 0.2	6.7 ± 0.3	0.357
TFM index (kg/m^2^)	6.9 ± 2.2	7.3 ± 2.6	7.6 ± 2.5	0.286	7.6 ± 0.2	7.4 ± 0.2	6.8 ± 0.2	0.065
VFA (cm^2^)	78.9 ± 30.4	80.9 ± 35.9	72.8 ± 36.4	0.371	92.8 ± 3.4	82.9 ± 2.6	57.2 ± 3.7^*^^#^	<0.001
nPNA (g/kg/day)	0.78 ± 0.21	0.85 ± 0.23	0.88 ± 0.17^*^	0.023	0.75 ± 0.03	0.85 ± 0.026^*^	0.92 ± 0.03^*^	0.006
GNRI	89.7 ± 9.7	94.2 ± 6.6^*^	97.1 ± 9.2^*^	<0.001	92.1 ± 1.4	94.9 ± 1.1	94.1 ± 1.5	0.223

*Data are expressed as the mean ± SD for univariate analysis or the mean ± SE for multivariate analysis. P-values were tested by a one-way ANOVA, followed by a Bonferroni post-hoc comparison on the univariate analyses and an analysis of covariance on the multivariate analyses. Multivariate analyses were adjusted for age, sex, the presence of diabetes mellitus, body mass index, urine volume, and edema index*.

*ALM, appendicular lean mass; TFM, total fat mass; VFA, visceral fat area; nPNA, normalized protein equivalent of total nitrogen appearance; GNRI, Geriatric Nutritional Risk Index*.

* *p < 0.05 compared with low tertile and*

# *p < 0.05 compared with middle tertile*.

### Comparison of Nutritional Indices According to PhAs

#### Prevalence of Sarcopenia

**Figure 2:**
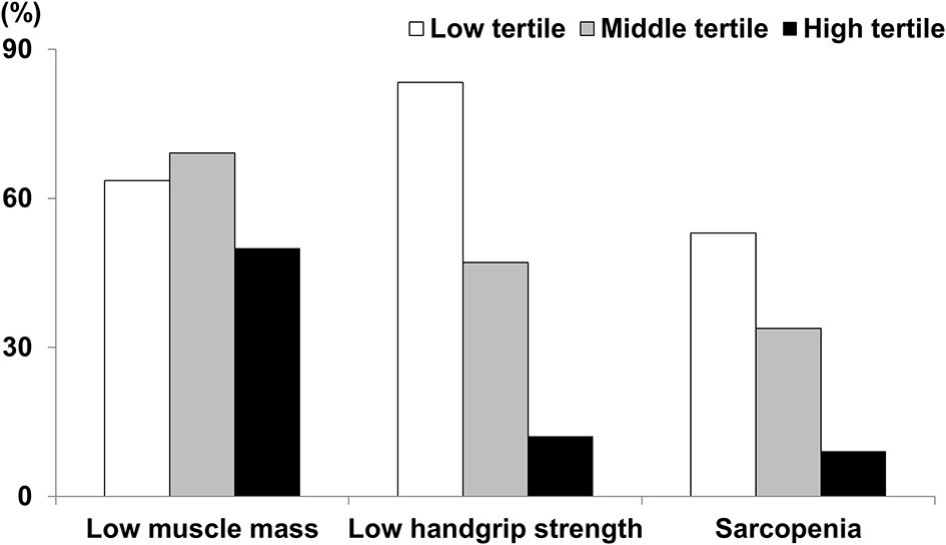
Proportions of patients with low muscle mass, low handgrip strength, or sarcopenia.

The number of patients with low muscle mass in the low, middle, and high tertiles was 42 (63.6%), 47 (69.1%), and 33 (50%), respectively (*p* = 0.067). The number of patients with low HGS in the low, middle, and high tertiles was 55 (83.3%), 32 (47.1%), and 8 (12.1%), respectively (*p* < 0.001). The number of patients with sarcopenia in the low, middle, and high tertiles was 35 (53%), 23 (33.8%), and 6 (9.1%), respectively (*p* < 0.001) (Figure 2). Among the three tertiles, the proportion of patients with low HGS or sarcopenia was highest in the low tertile. A multivariate logistic regression analysis showed that the patients in the low tertile had an odds ratio of 9.8 (*p* = 0.001) and 52.79 (*p* < 0.001) for developing sarcopenia compared with those in the middle and high tertiles, respectively (Table 3). The patients in the middle tertile had a 7.52 (*p* = 0.011) odds of developing sarcopenia compared with those in the high tertile.

**Table 3:** Logistic regression analysis for sarcopenia according to variables.

	**Univariate**		**Multivariate**	
	**Odds ratio (95% CI)**	***p***	**Odds ratio (95% CI)**	***p***
Age (per 1 year increase)	1.05 (1.02–1.08)	<0.001	1.04 (1.01–1.07)	0.023
Sex (ref: men)	1.15 (0.63–2.09)	0.651	0.43 (0.19–0.99)	0.046
Diabetes mellitus	1.36 (0.75–2.47)	0.315	1.28 (0.57–2.86)	0.553
BMI (per 1 kg/m^2^ increase)	0.80 (0.72–0.89)	<0.001	0.77 (0.67–0.89)	<0.001
Urine volume (per 1 ml/day increase)	1.00 (1.00–1.00)	0.335	1.00 (1.00–1.00)	0.795
Edema index (per 0.01 increase)	1.37 (1.08–1.74)	0.010	0.53 (0.32–0.87)	0.012
Tertile by phase angle				
Low tertile (ref: Middle tertile)	2.21 (1.10–4.44)	0.026	9.86 (2.49–39.02)	0.001
Low tertile (ref: High tertile)	11.29 (4.29–29.74)	<0.001	52.79 (8.84–315.19)	<0.001
Middle tertile (ref: High tertile)	5.11 (1.92–13.59)	0.001	7.52 (1.60–35.35)	0.011

*Multivariate analysis was adjusted for age, sex, presence of diabetes mellitus, body mass index, urine volume, and edema index*.

*CI, confidence interval; BMI, body mass index*.

#### AUROC of PhA for Sarcopenia

**Figure 3:**
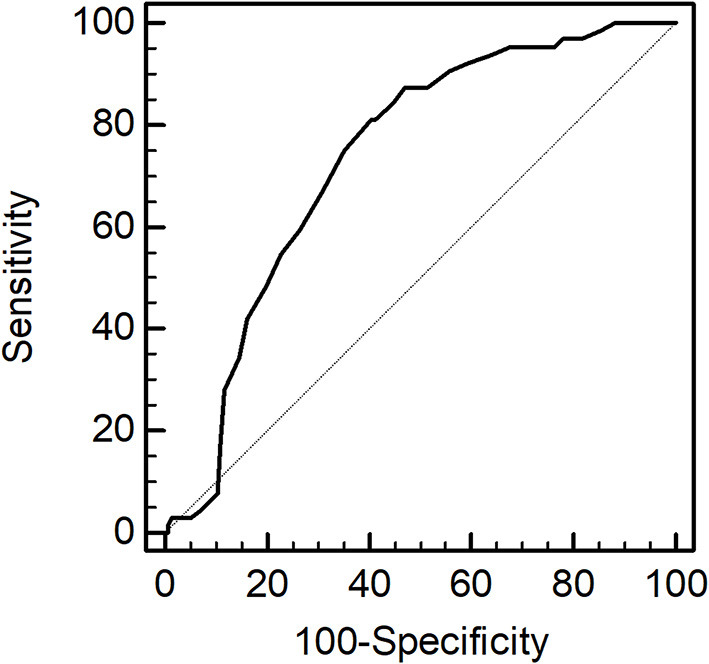
Receiver operating characteristic curve of phase angle used to predict sarcopenia.

The AUROC of PhA for sarcopenia was 0.73 (95% CI,0.67–0.79, *p* < 0.001, Figure 3). The sensitivity and specificity in predicting sarcopenia were 81.3% (95% CI, 69.5–89.9) and 59.6% (95% CI, 50.8–67.9), respectively. The optimal cut-off value was identified as ≤ 4.4°.

### Subgroup Analyses According to Age, Sex, and Presence of DM

We divided the patients into two age groups according to the median age of 55 years. For those aged <55 years, most of the variables except nPNA in the univariate analysis and GNRI in the multivariate analyses were significantly associated with PhA (Supplementary Table 3). In the multivariate analyses, all the variables except nPNA were statistically significant in those aged ≥55 years, with similar trends in those aged <55 years. In the analyses by sex or DM, all the variables except GNRI were associated with PhA in the multivariate analysis (Supplementary Tables 4, 5).

## Discussion

PhA is associated with the quantity of cell mass or cell membrane integrity (29). A high PhA reveals greater cellularity and relatively less water content to cell mass, which results in a low extracellular water/intracellular water ratio (30). Malnutrition caused by various diseases can lead to a shift from intracellular water to extracellular water and a decrease in cell mass. These changes result in a decrease in the level of PhA (30, 31). Therefore, previous studies have evaluated the association between PhA and malnutrition in various diseases or sarcopenia. This study also revealed that high PhA tertiles were associated with high HGS, total LM index, or nPNA. Patients with low PhA tertiles had greater sarcopenia, and PhA values, as continuous variables, were also correlated with HGS, total LM index, ALM index, and nPNA. Similar trends were obtained from linear regression analyses or subgroup analyses.

Previous studies have shown the association between PhA and nutritional status or sarcopenia under various conditions. Espirito Santo Silva et al. enrolled patients with liver cirrhosis and evaluated muscle mass by DXA and muscle strength using HGS (32). They showed an inverse association between PhA and sarcopenia and an angle ≤5.05° as a cutoff value for nutritional complication in patients with liver cirrhosis. Sarcopenia is originally defined as an age-related decline in muscle mass and is highly prevalent in the elderly population. Some studies on the elderly population showed a significant association between PhA and sarcopenia by muscle mass measurement (using ultrasonography or BIA) and HGS (33–35). Marini et al. evaluated an elderly population and showed a positive association between PhA and skeletal muscle mass index (36). However, muscle mass alone was evaluated by DXA. Two studies enrolled hospitalized patients and showed a positive association between PhA and sarcopenia combined with muscle mass using BIA, CT, and HGS (37, 38). Cancer is a well-known risk factor for sarcopenia. Pérez Camargo et al. evaluated palliative patients with cancer and revealed an association between PhA and muscle mass using a BIA (39). Souza et al. showed that PhA is a marker for muscle mass index, muscle density, and HGS in patients with colorectal cancer (40). Although kidney transplant recipients have favorable muscle mass or strength compared with patients undergoing dialysis, steroids or suboptimal renal function can lead to a negative balance of muscle mass (41). Two studies using kidney transplant recipients revealed a positive association between PhA and muscle strength or sarcopenia (42, 43).

Some studies evaluated the association between PhA and nutritional markers such as serum albumin or muscle mass measurements using a BIA in patients undergoing PD. Passadakis et al. showed a positive association between PhA and subjective global assessment as nutritional index in 47 patients undergoing PD (19). Fein et al. showed a positive correlation between PhA and albumin, total protein, and creatinine in 45 patients undergoing PD (20). Mushnick et al. enrolled 48 patients undergoing PD and showed that PhA is associated with patient survival and serum albumin level (21). A study from Korea enrolled 80 patients undergoing PD and showed that PhA was correlated with serum albumin, GNRI, and LM index from bioimpedance (22). However, in this study, muscle mass was evaluated using bioimpedance, and there were no data on muscle strength. Huang et al. enrolled a large sample of 760 patients undergoing PD and showed that low PhA is associated with high mortality, serum albumin, creatinine, and favorable residual renal function in patients undergoing PD (23).

The results of this study were similar to those from previous studies. PhA was associated with sarcopenia and/or each component of sarcopenia. The inverse association was stronger in muscle strength than muscle mass. We considered the effect of the volume on the LM index, and multivariate analyses were adjusted for edema index. In this study, PhA showed a different association with lean mass or fat mass. Although it is not well-known whether a high fat mass in patients undergoing dialysis is favorable, a high fat mass can be useful to prevent malnutrition. However, fat mass *per se* would be a hazard to overall cellular health and may be expressed as low PhA in patients undergoing PD.

In this study, we found no association between PhA and GNRI in the multivariate analysis. This may be caused by the strong association between GNRI and serum albumin. In particular, GNRI is calculated using body weight and serum albumin, which can be largely influenced by serum albumin (28). Serum albumin is a commonly used nutrition index, but it has many limitations in predicting nutritional status. It is also considered a negative acute phase protein and is inversely associated with volume status and inflammation. In addition, serum albumin may be normal in mildly malnourished patients because of hepatic adaptation according to malnutrition (44).

This study has several strengths. Several previous studies on sarcopenia have used incomplete criteria for sarcopenia, such as muscle mass alone. The muscle mass from previous studies has been estimated by anthropometry or with BIA or equations using creatine kinetics (19–23). Recent guidelines for diagnosing sarcopenia recommend both BIA and DXA as reasonable for assessing muscle mass (4, 5). However, patients undergoing PD have more volume overload than patients undergoing hemodialysis (45). A guideline suggests that performing DXA would be more reasonable than using a BIA to measure muscle mass in patients undergoing PD. First, this study enrolled patients who were undergoing PD alone as a single dialysis modality and included a relatively large sample size. Second, we used consensus definitions and cut-off values for Asian populations (5). Third, muscle mass measurements were evaluated by DXA, and sarcopenia was defined using two variables (low strength and low muscle mass). Considering the importance of the proper screening of sarcopenia in patients undergoing PD or the findings, PhA may be useful for deciding whether further evaluation is required to diagnose sarcopenia in patients undergoing PD.

This study has inherent limitations, namely, its single center and retrospective nature. It could not evaluate the causal relationship between variables and was not planned for a diagnostic study. A sample size calculation was not performed. However, owing to the relatively large sample size, the design of this study can be applied to evaluate the association between variables. Although this study showed an association between PhA and sarcopenia or sarcopenia-associated indicators in patients undergoing PD, it did not present the diagnostic efficacy of PhA for sarcopenia, such as sensitivity, specificity, and positive/negative predictive values. In addition, the use of PhA cannot easily diagnose sarcopenia because PhA value is not included in the diagnostic criteria for sarcopenia. Furthermore, the measurement of PhA requires a BIA machine, which is not available in all clinical settings. These findings reveal that the results of this study alone cannot provide sufficient evidence to predict or diagnose sarcopenia with PhA measurement. PhA could be considered an additional marker to suspect sarcopenia in patients at risk rather than a diagnostic property regarding these limitations. In addition, muscle mass measurement by DXA can be influenced by volume status. Baseline characteristics were significantly different among the three tertiles. Factors such as age, sex, and DM basically influence PhA, and patients with different PhAs will have different characteristics for these factors. However, we tried to attenuate these confounding factors by multivariate or subgroup analyses. A prospective longitudinal study that includes volume-independent muscle measurement and a larger number of patients is warranted to overcome these limitations.

This study demonstrated that PhA is independently associated with muscle mass, strength, and sarcopenia in patients undergoing PD. The results suggest that PhA can be used as a valuable and simple predictor for identifying patients undergoing PD who are at risk of sarcopenia.

## Data Availability Statement

The raw data supporting the conclusions of this article will be made available by the authors, without undue reservation.

## Ethics Statement

The studies involving human participants were reviewed and approved by IRB of Yeungnam University Medical Center (approval No: 2020-06-002). Written informed consent for participation was not required for this study in accordance with the national legislation and the institutional requirements.

## Author Contributions

SK conceptualized and designed the study, performed the data analysis and interpretation, and wrote the manuscript. AK and JD generated and collected the data. SK and JD drafted and revised the manuscript. All the authors approved the final version of the manuscript.

## Funding

This study was supported by the Medical Research Center Program (2015R1A5A2009124) through the National Research Foundation of Korea (NRF) funded by the Ministry of Science, ICT, and Future Planning. The funder had no role in the study design, the data collection, analysis, and interpretation, the manuscript writing, or the decision to submit the article for publication.

## Conflict of Interest

The authors declare that the research was conducted in the absence of any commercial or financial relationships that could be construed as a potential conflict of interest.

## Publisher's Note

All claims expressed in this article are solely those of the authors and do not necessarily represent those of their affiliated organizations, or those of the publisher, the editors and the reviewers. Any product that may be evaluated in this article, or claim that may be made by its manufacturer, is not guaranteed or endorsed by the publisher.
