# Supplementary material for: Association Between Phase Angle and Sarcopenia in Patients Undergoing Peritoneal Dialysis
Source: Front Nutr. 2021 Sep 24;8:742081. doi: 10.3389/fnut.2021.742081 (PMC8497817; doi:10.3389/fnut.2021.742081)
Supplement: Supplementary file 1 [file Data_Sheet_1.pdf]

Supplementary Table 1. Correlation between phase angle and various indices

	Univariate		Multivariate	
	r	***P*-value**	***r***	***P*-value**
Handgrip strength (kg)	0.616	<0.001	0.339	<0.001
Total LM index (kg/m^2)	0.284	<0.001	0.406	<0.001
ALM index (kg/m^2)	0.321	<0.001	0.251	0.001
Total FM index (kg/m^2)	0.170	0.016	–0.385	<0.001
VFA (cm^2)	0.002	0.981	–0.538	<0.001
nPNA (g/kg/day)	0.142	0.048	0.263	<0.001
GNRI	0.384	<0.001	0.100	0.173

Correlation analyses were analyzed using Pearson's correlation on univariate and partial correlation on multivariate. Multivariate analysis was adjusted for age, sex, the presence of diabetes mellitus, body mass index, urine volume, and edema index.

Abbreviations: *r*, correlation coefficient; LM, lean mass; ALM, appendicular lean mass; FM, fat mass; VFA, visceral fat area; nPNA, normalized protein equivalent of total nitrogen appearance; GNRI, geriatric nutritional risk index

Supplementary Table 2. Linear regression analysis of HGS or ALM index by variables

	**Univariate**		**Multivariate**	
	Standardized β (SE)	*P*-value	Standardized β (SE)	*P*-value
**Dependent variable: HGS**				
Age	–0.35 (0.05)	<0.001	–0.19 (0.04)	<0.001
Sex (ref: men)	–0.63 (1.50)	<0.001	–0.47 (0.89)	<0.001
Diabetes mellitus	–0.02 (1.26)	0.734	0.03 (0.89)	0.606
Body mass index	0.28 (0.16)	<0.001	–0.00 (0.12)	0.983
Urine volume	0.26 (0.00)	<0.001	0.04 (0.00)	0.458
Edema index	–0.43 (0.44)	<0.001	0.16 (0.68)	0.106
Phase angle	0.62 (0.05)	<0.001	0.56 (0.10)	<0.001
**Dependent variable: ALM index**				
Age	–0.19 (0.01)	0.007	–0.07 (0.01)	0.326
Sex (ref: men)	–0.26 (0.27)	<0.001	–0.08 (0.28)	0.256
Diabetes mellitus	0.03 (0.28)	0.726	0.01 (0.28)	0.929
Body mass index	0.41 (0.03)	<0.001	0.24 (0.04)	0.001
Urine volume	0.16 (0.00)	0.025	0.03 (0.00)	0.711
Edema index	–0.12 (0.11)	0.087	0.44 (0.21)	0.003
Phase angle	0.32 (0.01)	<0.001	0.58 (0.03)	0.001

Multivariate analysis was adjusted for age, sex, the presence of diabetes mellitus, body mass index, urine volume, and edema index.

Abbreviations: SE, standard error; HGS, handgrip strength; ALM, appendicular lean mass.

Supplementary Table 3. Correlation between phase angle and various indices by age

	<55 years (n = 95)				≥ 55 years (n = 105)			
	Univariate		Multivariate		Univariate		Multivariate	
	r	***P*-value**	***r***	***P*-value**	***r***	***P*-value**	***r***	***P*-value**
Handgrip strength (kg)	0.606	<0.001	0.453	<0.001	0.520	<0.001	0.204	0.047
Total LM index (kg/m^2)	0.324	0.001	0.498	<0.001	0.080	0.419	0.324	0.001
ALM index (kg/m^2)	0.304	0.003	0.238	0.028	0.213	0.029	0.281	0.006
Total FM index (kg/m^2)	0.330	0.001	–0.458	<0.001	0.014	0.886	–0.339	0.001
VFA (cm^2)	0.216	0.036	–0.497	<0.001	–0.176	0.073	–0.530	<0.001
nPNA (g/kg/day)	–0.002	0.986	0.306	0.004	0.318	0.001	0.182	0.076
GNRI	0.428	<0.001	–0.029	0.796	0.317	0.001	0.288	0.005

Correlation analyses were analyzed using Pearson's correlation on univariate and partial correlation on multivariate. Multivariate analysis was adjusted for age, sex, the presence of diabetes mellitus, body mass index, urine volume, and edema index.

Abbreviations: *r*, correlation coefficient; LM, lean mass; ALM, appendicular lean mass; FM, fat mass; VFA, visceral fat area; nPNA, normalized protein equivalent of total nitrogen appearance; GNRI, geriatric nutritional risk index.

Supplementary Table 4. Correlation between phase angle and various indices by sex

	Men (n = 114)				Women (n = 86)			
	Univariate		Multivariate		Univariate		Multivariate	
	r	***P*-value**	***r***	***P*-value**	***r***	***P*-value**	***r***	***P*-value**
Handgrip strength (kg)	0.627	<0.001	0.321	0.001	0.521	<0.001	0.442	<0.001
Total LM index (kg/m^2)	0.248	0.008	0.433	<0.001	0.010	0.926	0.380	0.001
ALM index (kg/m^2)	0.299	0.001	0.421	<0.001	0.316	0.003	0.291	0.009
Total FM index (kg/m^2)	0.171	0.070	–0.451	<0.001	0.342	0.001	–0.313	0.005
VFA (cm^2)	–0.013	0.892	–0.643	<0.001	0.114	0.295	–0.437	<0.001
nPNA (g/kg/day)	0.192	0.044	0.292	0.003	0.142	0.197	0.240	0.033
GNRI	0.451	<0.001	0.065	0.509	0.275	0.010	0.111	0.330

Correlation analyses were analyzed using Pearson's correlation on univariate and partial correlation on multivariate. Multivariate analysis was adjusted for age, the presence of diabetes mellitus, body mass index, urine volume, and edema index.

Abbreviations: *r*, correlation coefficient; LM, lean mass; ALM, appendicular lean mass; FM, fat mass; VFA, visceral fat area; nPNA, normalized protein equivalent of total nitrogen appearance; GNRI, geriatric nutritional risk index.

Supplementary Table 5. Correlation between phase angle and various indices by the presence of diabetes mellitus

	Non-diabetes mellitus (n = 101)				Diabetes mellitus (n = 99)			
	Univariate		Multivariate		Univariate		Multivariate	
	r	***P*-value**	***r***	***P*-value**	***r***	***P*-value**	***r***	***P*-value**
Handgrip strength (kg)	0.708	<0.001	0.305	0.003	0.527	<0.001	0.386	<0.001
Total LM index (kg/m^2)	0.608	<0.001	0.575	<0.001	0.074	0.468	0.240	0.021
ALM index (kg/m^2)	0.412	<0.001	0.326	0.002	0.169	0.094	0.310	0.003
Total FM index (kg/m^2)	0.233	0.019	–0.498	<0.001	0.131	0.197	–0.310	0.003
VFA (cm^2)	0.123	0.219	–0.528	<0.001	–0.029	0.772	–0.564	<0.001
nPNA (g/kg/day)	0.088	0.391	0.325	0.002	0.228	0.024	0.225	0.031
GNRI	0.390	<0.001	0.129	0.222	0.387	<0.001	0.064	0.544

Correlation analyses were analyzed using Pearson's correlation on univariate and partial correlation on multivariate. Multivariate analysis was adjusted for age, sex, body mass index, urine volume, and edema index.

Abbreviations: *r*, correlation coefficient; LM, lean mass; ALM, appendicular lean mass; FM, fat mass; VFA, visceral fat area; nPNA, normalized protein equivalent of total nitrogen appearance; GNRI, geriatric nutritional risk index.
